# Evaluation of PLA and PETG as 3D-Printed Reference Materials for Compressive Strength Testing

**DOI:** 10.3390/ma18163794

**Published:** 2025-08-13

**Authors:** Bartosz Budziński, Karol Federowicz

**Affiliations:** Faculty of Civil and Environmental Engineering, West Pomeranian University of Technology in Szczecin, 70-310 Szczecin, Poland; bartosz.budzinski@zut.edu.pl

**Keywords:** reference materials, 3D printing, PLA, PETG, compressive strength, aging, quality control, construction material testing

## Abstract

This study explores the feasibility of using 3D printing technology to fabricate reference materials for validating compressive strength measurements in construction laboratories. Polylactic acid (PLA) and polyethylene terephthalate glycol-modified (PETG) were selected due to their widespread availability and use in fused deposition modeling (FDM). A series of cubic samples with varying infill levels and dimensions were printed and tested to evaluate the influence of infill density, temperature, and storage time on compressive strength. PLA samples exhibited higher compressive strength values (from 23.5 kN for 10% infill to 70.7 kN for 50% infill) and a steeper increase in strength with rising infill density compared to PETG (from 12.4 kN for 10% infill to 44.1 kN for 50% infill). However, PETG demonstrated superior stability over time, with significantly smaller increases in result variability after 31 days. The results confirm a strong linear correlation between infill level and compressive strength and indicate that even small fluctuations in ambient temperature can influence test outcomes. Despite PLA’s initial mechanical advantage, PETG’s aging resistance makes it a promising candidate for the development of durable and repeatable reference materials (increment of StD for PLA from 0.17 kN to 0.63 kN and 0.25 kN to 0.37 for PET-G). This research contributes to closing the gap in the availability of reliable mechanical reference materials for destructive testing, offering a novel application for 3D printing in quality control in civil engineering.

## 1. Introduction

Equipment control in the laboratory is a crucial process in managing a research unit. Adequate supervision is one of the requirements of the international standard ISO/IEC 17025:2017 [1,2]. Equipment used for measurements must ensure the required measurement accuracy and/or measurement uncertainty necessary to obtain a valid result (Section 6.4.5 of the standard [2]). Validation of results can be achieved in several ways, one of which is the use of reference materials or quality control materials. In the case of compressive strength presses used in construction laboratories, obtaining the appropriate reference material is problematic. Reference materials are regularly used in chemical and medical laboratories [3,4]. A reference material should exhibit appropriate stability [5]. Such reference materials for cement-bound mixtures are not commercially available. On the other hand, retesting of stored items is not feasible (Section 7.7.1.g of the standard), as destructive testing is conducted in construction laboratories and the materials tested are composites containing cement as a binder, thus ensuring the appropriate stability of the tested object over time is not possible. There are, indeed, reference materials for cement testing; however, they relate to the chemical composition of the cement [6] as well as the rheological properties of the cement paste [7]. Therefore, there is a demand for technical solutions that allow for quick and simple verification by the laboratory’s intermediate staff. One such method could be the use of 3D printing technology. This technology has been widely implemented in prototyping as well as in the production of plastic components [8]. Additive manufacturing also utilizes other materials as filaments, including concrete, clay, or metal powders [9,10]. In this publication, we present preliminary results from the use of 3D printing to prepare reference materials that can be used in a construction laboratory to validate the results.

Despite the widespread use of 3D printing in prototyping and component manufacturing, its application in creating reference materials for strength testing has not yet been described. This is mainly due to the lack of data on the long-term mechanical stability of printed elements and the influence of environmental conditions. Limited results concerning the effect of aging on the compressive strength of PLA elements can be found in the study by Rimkus et al. [11], who suggest that material stored under laboratory conditions retains its strength even after 200 days, with only a reduced ability to undergo plastic deformation. These findings contradict the results obtained by Nallamuthu et al. [12], who demonstrated significant aging of elements over time when exposed to moisture. Meanwhile, Mitrović et al. [13] showed that the bending strength of PLA samples aged for two weeks under room conditions can increase by almost 9%.

This article aims to address this gap by systematically analyzing the impact of geometry, temperature, and storage time on the strength of PLA samples. The available literature data are inconsistent, and the boundary conditions described do not reflect standard sample storage conditions in a construction laboratory. The novelty of the presented research lies not in the use of 3D printing itself, but in its application for producing low-cost reference materials intended for routine compressive strength testing, with a comprehensive comparison of factors affecting their performance and repeatability. In the first stage of the study, the focus was placed on determining the relationship between the infill density of the samples and their compressive strength. The next step involved evaluating the effect of temperature on strength at a constant infill level and assessing the aging resistance of samples made from PLA and PET-G.

## 2. Materials and Methods

**Figure 1:**
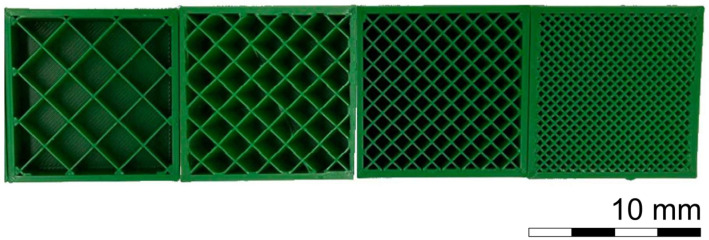
Comparison of the fill levels of individual cubes from 10 to 50%.

Reference material in the form of cubic samples with varied infill levels and dimensions was produced using 3D printing technology. A cross-section illustrating the infill method and size of the samples is shown in Figure 1. For the tests, polylactic acid (PLA) and polyethylene terephthalate glycol-modified (PETG) was selected as the filament [14]. The polymer parameters are presented in Table 1.

PLA was chosen as it is a biodegradable polymer [17], derived from renewable resources, while PETG is one of the most commonly used filament due to its superior mechanical properties. Polylactic acid (PLA) was selected for the study not only due to its origin from renewable resources and the absence of harmful emissions during printing, but also because of its wide availability, low cost, and favorable mechanical properties when printed using inexpensive, commercially available 3D printers. PLA is also characterized by low shrinkage, which reduces the risk of sample deformation and improves result repeatability. Compared to other polymers such as ABS or nylon, PLA is less demanding in terms of printing conditions (e.g., it does not require an enclosed chamber or high bed temperatures), making it an ideal material for preliminary studies on reference materials for strength testing presses. The second polymer selected for the study was polyethylene terephthalate glycol-modified (PETG), chosen due to its widespread use in home 3D printing. The cubes were produced using a Bambu Lab A1 printer (Bambu Lab, Shenzhen, China), utilizing a standard 0.4 mm nozzle. The PLA and PETG used in the studies came from a single batch, was factory-sealed, and the material had a diameter of 1.75 mm. Three different sizes of cubes were designed and manufactured—2, 3, and 4 cm—to assess the influence of size effect on the strength and stability of the reference material. The printing parameters are presented in Table 2 and were adjusted based on the filament manufacturer’s recommendations. Incorrectly selected printing parameters can lead to excessive deformation of the element, rounding of edges, and in extreme cases, even delamination [18].

An open-chamber printer was used, which was in a laboratory room with constant temperature conditions (T = 20 °C, RH = 55%). The cubic samples were made with four full layers (outlining the walls, bottom, and top surfaces), and a mesh infill ranging from 10 to 50% of the sample volume, as shown in Figure 1. The infill effect was evaluated using 4 × 4 × 4 cm cubes. Short-term aging tests were performed after 31 days on 4 × 4 × 4 cm cubes with 25% infill.

To estimate the impact of various factors, several series of tests were conducted. The influence of the infill level and the size of the tested cubes was examined. These two characteristics will determine the force obtained upon destruction, and thus, based on them, it will be possible to design appropriate reference materials. Considering that the tested material is a polymer, the effect of temperature on the force obtained upon destruction was determined. This will dictate how to handle the reference material. Each series involved testing 6 samples. The following variables were tested: infill level (10, 25, 35, 50%), test temperature (10, 20, 30 °C), and cube dimensions (2, 3, 4 cm). The selection of this temperature range was dictated by the need to reflect the possible conditions in the construction laboratory. Samples were placed in a temperature-controlled chamber with a precision of ±0.5 °C for 4 h prior to testing. The temperature was continuously monitored using calibrated thermocouples to ensure consistency throughout the conditioning period. In addition, an assessment of the stability of the tested objects over time was conducted, which allowed for the determination of their durability and possible changes in parameters during storage. Due to the preliminary nature of the work, stability was determined one month after the samples were made. The samples for stability assessment were stored without access to air and light at a temperature of 20 °C. All samples were weighed and measured before testing to confirm the repeatability of the prints. The testing was conducted in a compressive strength press (Contols Uniframe UTM250, The CONTROLS Group, Milan, Italy) with a constant force increase of 1.0 kN/s, after all samples were conditioned at the test temperature for 4 h. For the analysis of results, linear regression, the *t*-test, and box-and-whisker plot analysis were used.

## 3. Results and Discussion

**Figure 2:**
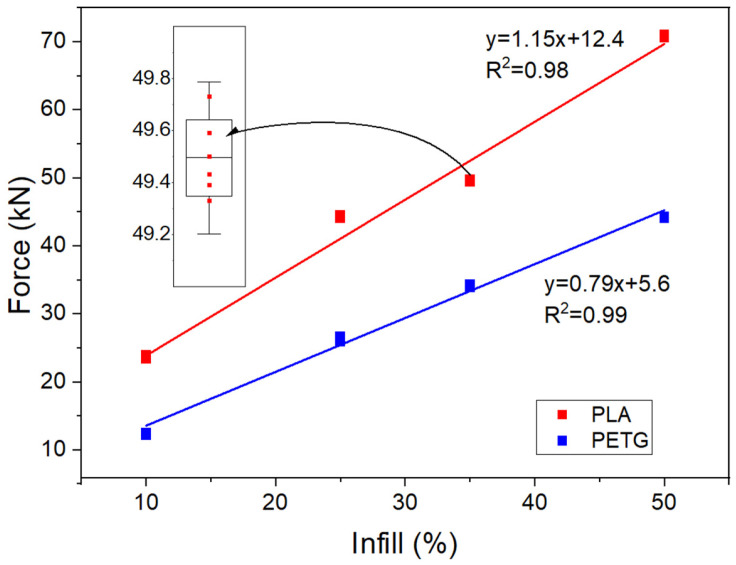
Destructive force results obtained for different infill levels.

**Figure 3:**
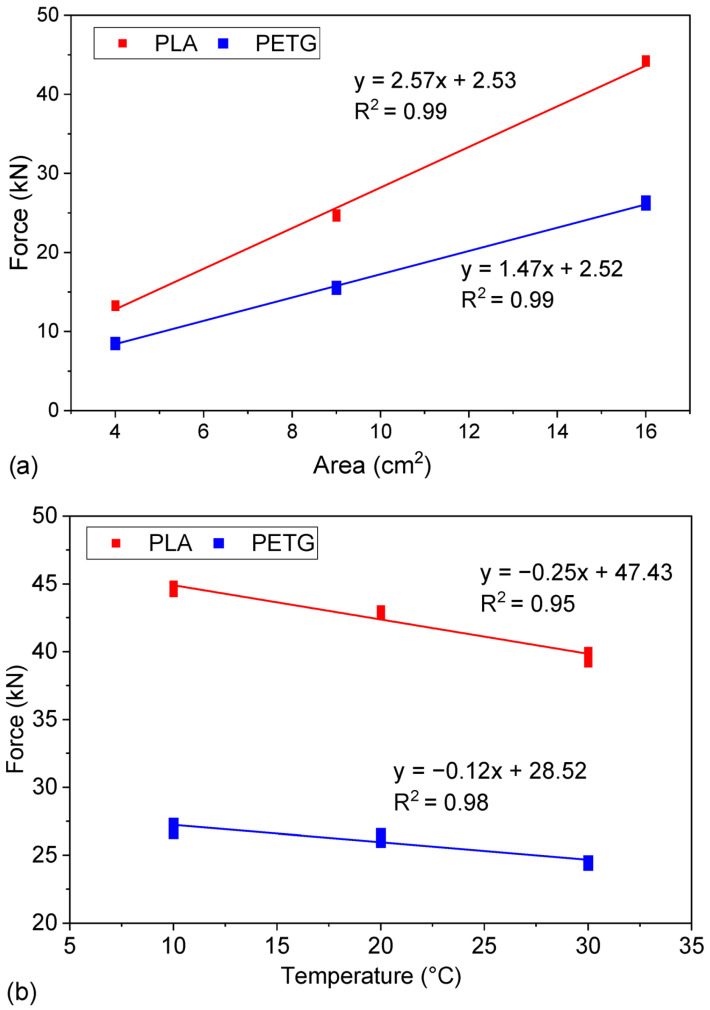
Results obtained for variables: (**a**) surface area of the cube at 25% infill level; (**b**) test temperature.

Based on the conducted compressive strength tests (PLA and PETG), it must be stated that there is a very strong linear relationship between the infill level (Figure 2) and the destructive force, as well as the surface area of the tested cube (Figure 3a) and the destructive force. This indicates that neither the size nor the manufacturing method of the reference material affects its strength, unlike in the case of cement materials. Attention should be paid to the very high repeatability of the results. The points on the chart are so close to each other that individual results cannot be distinguished. Only by enlarging the chart is it possible to observe individual results. Such an outcome for potential reference material is highly desirable. It allows for the preparation of a reference material with very low uncertainty, which is advantageous when confirming the validity of test results and controlling equipment. In the case of PLA, the obtained strength results are significantly higher than those of PETG. Additionally, the slope coefficient is greater, indicating a faster increase in force with the rising infill level.

The relationship between the sample surface area and the compressive force is linear (Figure 3a). This makes it easy to prepare reference materials with the desired compressive strength by appropriately scaling samples. Since PLA and PETG are thermoplastic materials, the temperature at which research samples are stored affects their strength. Using the determined linear relationship between temperature and destructive force (Figure 3b), even within a small range, such as from 19 to 21 °C, the change in destructive force would be 0.51 kN, which represents about 1.2% of the value. In the case of PETG, within the temperature range of 19 to 21 °C, the change in destructive force is only 0.25 kN, which represents approximately 0.95% of the mean value. Therefore, the use of reference materials made from PLA and other plastics used in 3D printing technology requires not only precise temperature control during tests but also appropriate conditioning of the samples before testing. Despite considering the impact of temperature, the spread of results remains significantly smaller than those observed in studies of materials based on cement binders. This indicates the potential of 3D printing technology in creating stable and repeatable reference materials that can be successfully used in construction laboratories.

**Figure 4:**
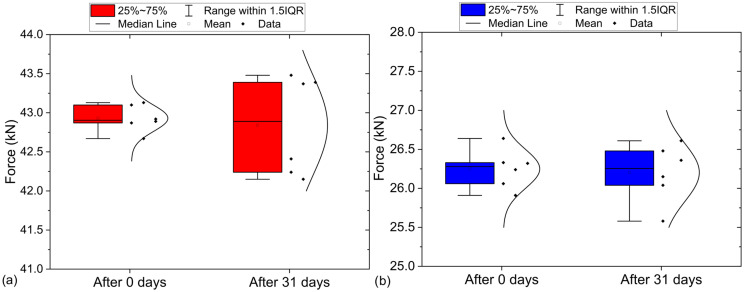
Stability test results after 0 days and after 31 days: (**a**) PLA and (**b**) PETG.

Figure 4 presents a comparison of the destructive force results for PLA and PETG samples tested immediately after printing (0 days) and after 31 days of storage under controlled conditions (20 °C, no access to air or light). Samples examined immediately after printing and after 31 days showed no visible differences, either in organoleptic assessment or under an optical microscope. While Student’s *t*-test indicated no statistically significant differences (PLA *p*-value = 0.7441 ≥ 0.05 PETG *p*-value = 0.8304 ≥ 0.05), the box-and-whisker plot reveals a notable increase in the spread of results after 31 days (PLA). Since in the case of PLA, the spread of results was greater after 31 days than immediately after sample preparation, the range increased from 0.46 to 1.33 kN, while the standard deviation rose from 0.17 to 0.63 kN. The obtained results partially confirm the findings of Rimkus et al. [11]. In terms of stability, PETG demonstrated significantly better properties. The increased variability in PLA is most likely the result of material aging, which in turn leads to lower precision in the test results.

## 4. Conclusions and Future Remarks

This study demonstrates the feasibility of using 3D printing to develop standardized reference materials for compressive strength testing in construction laboratories. By employing commonly available filaments—PLA and PETG—cubic samples were produced and assessed under various infill densities, temperatures, and storage conditions. Based on the studies conducted and analyses, the following conclusions were formulated:

- Three-dimensional-printed polymers can serve as reliable reference materials for compressive strength testing, addressing the current gap in commercially available reference solutions for cement-bound mixtures used in destructive testing. PLA showed higher initial compressive strength.
- PETG demonstrated superior long-term dimensional and mechanical stability, with minimal variability in test results after aging. This positions PETG as a more appropriate candidate for repeatable and reusable reference samples.
- Linear relationships between compressive force and both infill density and sample size allow for precise calibration of reference strength values through controlled geometry scaling.
- Even small fluctuations in test temperature (±1 °C) can affect compressive strength outcomes, especially for PLA. Therefore, strict environmental control and sample conditioning are essential to ensure measurement accuracy.
- The high repeatability of results for both materials—exceeding typical consistency for cementitious samples—highlights the potential of 3D printing in developing low-uncertainty reference standards for internal quality control.
- The printing times for PLA and PETG samples are nearly identical, and with material costs of approximately EUR 17/kg for PLA and EUR 19/kg for PETG, the overall cost difference is negligible.

## Figures and Tables

**Table 1:** The physical and mechanical properties of the poly-lactic-acid filament [15,16].

Property	Value	
	PLA	PET-G
Full Name	Polylactic acid	Polyethylene terephthalate glycol-modified
Melting Point	150–160 °C	160–293 °C
Glass Transition	60–65 °C	79–85 °C
Density	1.210–1.430 g·cm^−3^	1.26–1.28 g·cm^−3^
Tensile Modulus	2.7–16 GPa	1.90–2.00 GPa

**Table 2:** Print parameters.

Property	Value	
	PLA	PET-G
Printing temperature	220 °C	235 °C
Bed temperature	55 °C	70 °C
Nozzle	0.4 mm	
Layer height	0.2 mm	
Infill pattern	Rectilinear	
Infill density (%)	10, 25, 35, 50%	

## Data Availability

The original contributions presented in this study are included in the article. Further inquiries can be directed to the corresponding author.
